# Abrasive Wear of Mining Chain Drums Made of Austempered Ductile Iron in Different Operating Modes

**DOI:** 10.3390/ma15082709

**Published:** 2022-04-07

**Authors:** Andrzej N. Wieczorek, Mateusz Wójcicki, Andrzej Drwięga, Waldemar Tuszyński, Paweł M. Nuckowski, Jakub Nędza

**Affiliations:** 1 Faculty of Mining, Safety Engineering and Industrial Automation, Silesian University of Technology, Akademicka 2 Street, 44-100 Gliwice, Poland; 2 Division of Machines and Equipment, KOMAG Institute of Mining Technology, Pszczyńska 37 Street, 44-101 Gliwice, Poland; mwojcicki@komag.eu (M.W.); adrwiega@komag.eu (A.D.); 3 Tribology Center, Lukasiewicz Research Network-Institute for Sustainable Technologies (L-ITEE), ul. Pulaskiego 6/10, 26-600 Radom, Poland; waldemar.tuszynski@itee.lukasiewicz.gov.pl; 4 Materials Research Laboratory, Faculty of Mechanical Engineering, Silesian University of Technology, Konarskiego 18A Street, 44-100 Gliwice, Poland; pawel.nuckowski@polsl.pl; 5 Patentus SA, ul. Górnośląska 11, 43-200 Pszczyna, Poland; j.nedza@patentus.pl

**Keywords:** mining, wear, scraper conveyors, austempered ductile iron

## Abstract

The paper presents results of testing the resistance of chain wheels made of alloyed austempered ductile iron (ADI) with various content of retained austenite and subjected to shot peening, to the dynamic and abrasive wear by solid particles. The impact of the additional environmental factor—external dynamic forces—accompanying the operation of the chain wheels in the presence of the quartz particles has a synergistic effect on the abrasive wear in the contact area between the wheels and the chain links for all the considered variants, except for the ADI with the structure of the upper ausferrite. Based on the results obtained, it was found that the abrasive wear by solid particles increased and that the hardness of the surface layer of the chain wheels subjected to shot peening decreased. The relative increase in the wear ΔV_DYN_/δ_MAX,(A)_, representing the share of the additional dynamic force in the process of wear, was in the range of 16–40% for the group of tested cast iron ADI not subjected to shot peening, while for the shot peened—in the range of 16–64%. Demonstration of phase changes during the operation of chain wheels and the change in their intensity depending on the combination of environmental factors is the work novelty. In the opinion of the authors, the presented results will be of great practical importance as they will allow to increase the knowledge on the proper selection of ADI cast iron for environmental conditions.

## 1. Introduction

**Figure 1:**
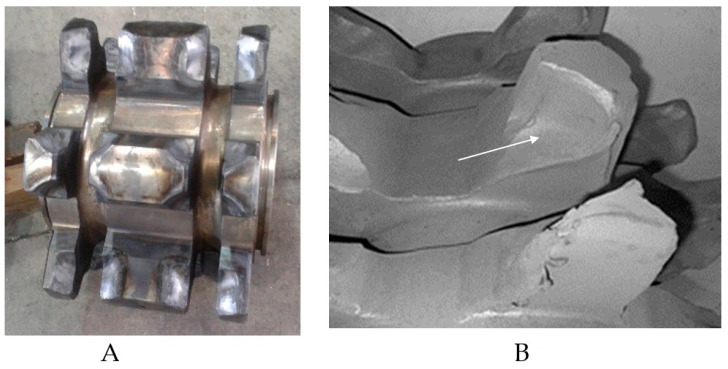
Chain wheel. (**A**) View of not damaged chain drum. (**B**) Damage to chain drums caused by abrasive wear by solid particles; the wear area is indicated by an arrow.

In the case of machines’ components for the exploitation of energy resources in harsh environmental conditions, e.g., presence of hard grains of minerals and salty waters, premature wear is observed. It is caused, among other factors, by the impact of the aggressive abrasive particles [1,2,3,4,5,6,7]. An example of a friction pair that is particularly vulnerable to wear is the following friction pair: seat of the chain wheel (Figure 1A) of armored face conveyor—chain link. This tribological system is subjected to particularly intensive wear during the operation (Figure 1B), which leads to damage preventing the correct operation of these machines.

A natural expectation of users of machines is to increase the durability and wear resistance of the components subjected to wear. Austempered ductile iron (ADI) is a material that can replace forged alloy steels subject to surface hardening in the construction of chain drums used in mining.

In surface-hardened steels and cast steel, materials which are used for construction of chain drums, the features characterizing the surface layer change in a gradient manner depending on the distance from the surface h more detail in the paper [8]. 

The degressive character of the course of the parameters responsible for the wear resistance, such as microstructure or hardness, as a function of the distance from the surface, results in a variable intensity of wear of the surface layer during the operation.

Austempered Ductile Iron (ADI) is subjected to special heat treatment in the form of isothermal quenching. As a result of this type of treatment, cast iron acquires special properties in the form of a high yield point with relatively good plastic properties, which results in good impact toughness with high hardness. The starting nodular iron for ADI production most often contains Mo, Ni and Cu in order to increase the hardenability and plasticity.

Austempered ductile iron shows a tendency to surface hardening as a result of the phase transformation of austenite [9,10,11,12] into martensite under the load. This phenomenon was described in [13,14,15,16]. 

The available literature includes a large number of scientific studies on wear properties of cast iron subject to isothermal treatment, in which it has been found that ADI has a high resistance to abrasive wear [17,18,19]. In some cases, the wear resistance of ADI was equal to the wear resistance of surface hardened alloy steel [20]. As shown in the aforementioned publications [17,18,19], the favorable wear resistance properties of austempered ductile iron result from the phase transformation of austenite into martensite under the load.

The occurrence of the transformation of austenite into martensite in the surface layer of chain wheels made of ADI significantly changes the properties of the surface layer of the ADI and, thus, modifies its wear resistance. This affects the distribution of properties of ADI depending on the distance from the surface. The austempered ductile iron, in the non-deformed state, should have nearly the same tribological properties in the entire cross-section, but as a result of the transformations, ADI should be treated as a gradient material, the characteristic parameters of which depend on the distance h and load N. 

As it has already been mentioned, martensite is formed, which improves the wear resistance, but at the same time, increases the susceptibility of the surface layer to cracking. Putatunda & Bingi [21] showed that along with the decrease in the austempering temperature, and consequently a decrease in the content of austenite, the fracture toughness of ADI was reduced (represented by the parameter K_1C_). This relationship was found also by Yang & Putatunda [22] and, in the range of austempering temperatures of 280–380 °C, by Ravishankar et al. [23].

Abrasive-dynamic wear is a type of damage often encountered during an operation of machines such as crushers, armoured face conveyors and skip hoists. It combines the action of hard abrasive (e.g., quartz) and an external dynamic load. The expected combined action of both factors, intensifying the destructive processes, should cause an effect of synergy that can be presented in the following form:

(1) $$W_{T}=W_{ABR}+\Delta W_{DYN}$$

where *W_T_*—total wear, *W_ABR_*—abrasive wear, Δ*W_DYN_*—synergistic component related to dynamic loads.

After a simple conversion, the synergistic component Δ*W_DYN_* related to the action of dynamic forces can be determined from the formula:

(2) $$\Delta W_{DYN}=W_{T}-W_{ABR}$$

In order to determine the interactive component Δ*W_DYN_*, two series of wear tests are required—one in the conditions of abrasive wear and the other one in the conditions of combined action of the abrasive and the external dynamic force. As a part of this project, both wear tests were conducted. 

The scope of the work also included an analysis of impact of the surface dynamic treatment (shot peening) on the total wear in the area of cooperation between the chain wheel and the chain links.

In [24,25,26], a positive impact of shot peening on the characteristics of the toothed components were observed especially an increase in the fatigue strength and surface hardness. Introduction of advantageous compressive residual stresses to the surface layer was also observed. However, the preliminary studies [27], concerning the impact of shot peening on the abrasive wear in the area of cooperation between the chain wheels and the chain, did not confirm such an advantageous impact of shot peening.

Summarizing the presented state of the art, it is stated that there is a large operational problem with chain wheels used in the mining industry, potential possibilities of using the properties of ADI cast irons for manufacture of said chain wheels and contradictory results in terms of the impact of shot peening on the wear resistance of ausferritic cast iron. 

Novelty of the work, in relation to other projects, is the demonstration, in operational conditions, of the ability of chain wheels made of ADI cast irons to strengthen in the result of phase changes and finding the significant impact of environmental conditions, such as dynamic load, on their intensity. Moreover, the work presents synergistic components for the tested technological and operational associations related to the action of dynamic forces.

## 2. Materials and Methods

### 2.1. Characteristics of the Tested ADI

EN-GJS-600-3 (PN-EN 1563) ductile iron of the composition presented in Table 1 was the input material for making the ADI. In the pearlitic-ferritic structure of nodular iron, the graphite nodule count was 200 per 1 mm^2^, while the spheroidization of graphite was greater than 90%.

Parameters of heat treatment in salt bath are given in Table 2, while the mechanical properties—in Table 3. After the wear tests, metallographic tests and measurements of hardness were carried out. Brinell HB hardness was measured in the cross-section of the area of mating surfaces between the bottom of the tooth seat and the chain link. Samples for microstructure examinations were also cut from this area. Then, the samples were ground, polished and etched using the 2% Nital solution. A Zeiss AXIO Observer optical microscope (Oberkochen, Germany) was used for microscopic observations. The following scanning electron microscopes: HITACHI S-3500N (Tokyo, Japan) and Supra 35 ZEISS (Oberkochen, Germany) with EDS spectrometer were used for observations of the mating surfaces. The surface hardness was measured [28] by the Vickers method (the results for comparison were converted to HB).

**Figure 2:**
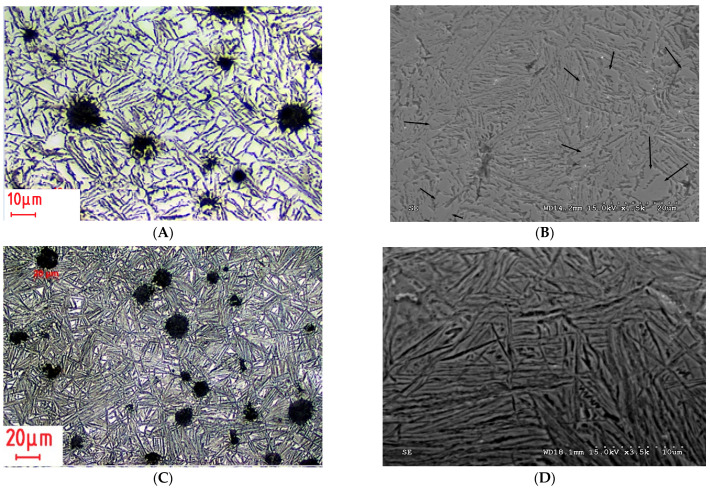
Views of the structure of the tested cast irons. (**A**) The microstructure of the ADI_360 matrix with the visible structure of upper ausferrite (OM). (**B**) Block austenite (marked by arrows) in the ADI_360 matrix (SEM). (**C**) The microstructure of the ADI_310 matrix (OM). (**D**) The microstructure of the ADI_240 matrix (SEM).

Based on tests of the microstructure, it was found that for the ADI austempered at the temperature of 360 °C (ADI_360) the upper ausferrite, composed of bainitic ferrite and austenite, was found in the matrix structure (Figure 2A). Block austenite was also found in this ADI structure of (Figure 2B). The total content of austenite was estimated at the level of 40%. In the case of the ADI_310 matrix, upper ausferrite (Figure 2C) with the austenite content of approx. 27% was found. In the case of this ADI, presence of block austenite was also identified. The austenite content was estimated on the basis of microstructure analysis and confirmed by XRD tests. 

The structure of the ADI_270 and ADI _240 matrix (sample view of the structure with SEM is shown in Figure 2D) consists of lower ausferrite composed of ferrite, traces of martensite and high-carbon austenite, the content of which in these ADI samples was at the level of 20% and 17%, respectively. 

**Figure 3:**
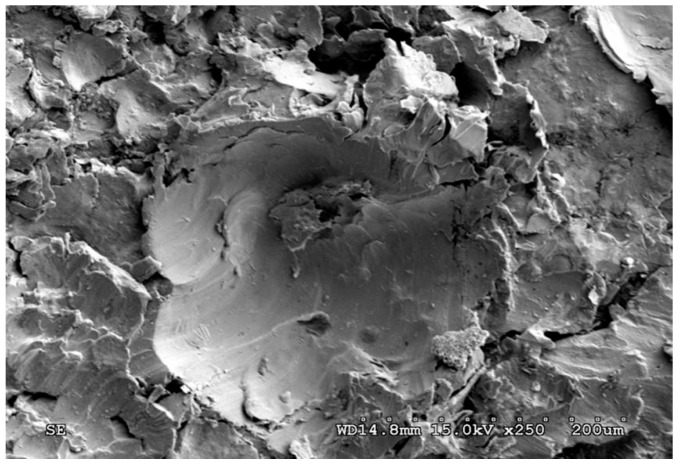
Surface of ADI_310_SP after shot peening (SEM).

**Figure 4:**
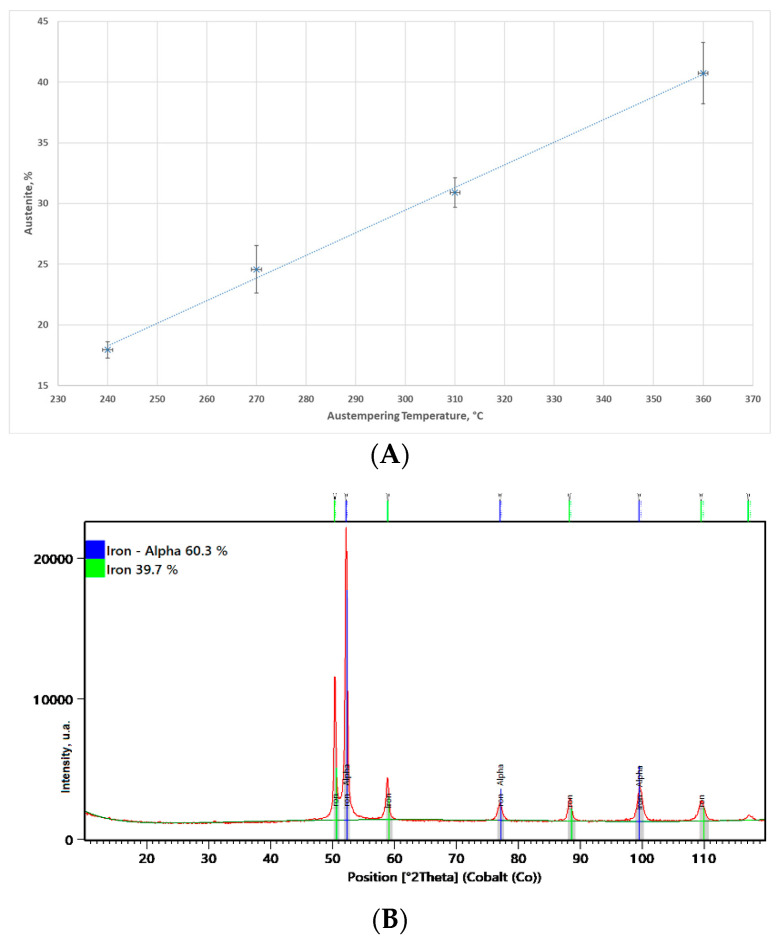
Austenite content. (**A**) Graph of the austenite content in the tested ADI matrix in a function of the austempering temperature. (**B**) Sample of X-ray diffractogram for ADI_360 cast iron.

A view of the surface of the chain wheel made of ADI_310_SP (after shot peening) is presented in Figure 3. As a result of the impact of the shot on the teeth surface, spherical deformations and cracks in the surface layer were revealed. Figure 4A shows a graphical presentation of the content of austenite in the matrix of the ADI tested as a function of the austempering temperature. An almost linear correlation between these parameters can be observed. 

Figure 4B shows a sample X-ray diffraction pattern for ADI_360 cast iron. XRD tests were made on laboratory samples in the X’Pert PRO MPD X-ray diffractometer by Panalytical (Almelo, The Netherlands) equipped with a cobalt anode X-ray tube (λKα = 0.179 nm) and a PIXcel 3D detector (Almelo, The Netherlands) on the axis of the diffracted beam. The diffractograms were recorded in Bragg-Brentano geometry in the angle range of 10–120 °2Theta with a step of 0.05° and a count time per step of 40 s.

The test samples were cut from the area of the seats from an additional series of chain wheels that were not subjected to tribological tests. They were cube-shaped with a side of 10 mm. The quantitative share of the Fe alpha and Fe gamma crystalline phases in the tested materials was calculated using the Rietveld method. The calculations were made in the dedicated HighScore Plus software (v. 3.0e) (Almelo, The Netherlands) with the use of the PAN-ICSD structural file database (Karlsruhe, Germany).

### 2.2. Characteristics of the Testing Methodology

**Figure 5:**
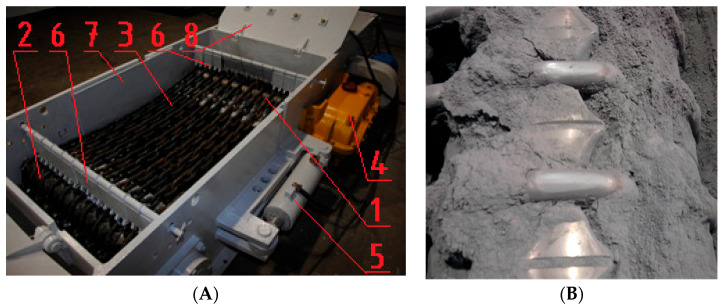
Test rig. (**A**) Rig (1—drive wheels, 2—turning wheels, 3—chains, 4—drive, 5—tensioner, 6—beaters, 7—main frame, 8—cover). (**B**) Testing chain drums filled with dry quartz abrasive.

Alloy cast iron was tested for wear properties in a test rig designed especially for that purpose, which allowed reproducing of real operating conditions of chain wheels. The test rig is presented in Figure 5A, while the detailed methodology and the test rig itself were described in [29]. The abrasive wear by solid particles of the chain wheels was reproduced by filling the test box with quartz particles (Figure 5B), which resulted in a presence of the abrasive material between the chain wheels and the chain surface all the time. In turn, the dynamic load was reproduced by suspending the beaters (Figure 5A) which, in a result of drum movement, hit the teeth bases. 

**Figure 6:**
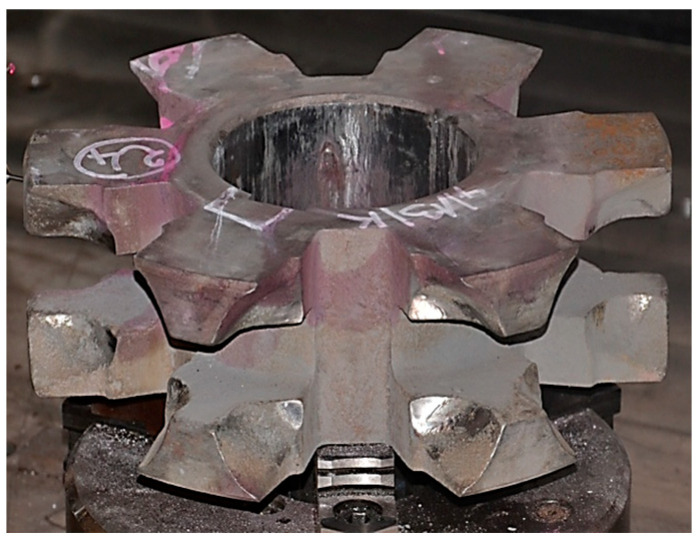
Tested chain wheel.

Two identical sets of chain wheels were used for the wear tests (Figure 6). One of them was subjected to shot peening (ADI_240_SP, ADI_270_SP, ADI_310_SP and ADI_360_SP), while the other one was not (ADI_240, ADI_270, ADI_310 and ADI_360) (explanation of material suffixes in Table 4).

The chain wheels were subjected to a dynamic treatment (shot peening) with the Almen intensity of 0.32 mmA and a coverage of 2 × 100% in the area of contact between the wheel and the chain. Cut and rounded shot of the diameter 0.6 mm and the hardness of approx. 54 HRC was used in shot peening. 

Each wear test lasted 200 h, 100 h for each direction of the motor’s rotation. The tangential velocity of the chain wheels was v = 0.7 m/s, while the power of the motors was 7.5 kW. The surface pressures determined between the surface of the wheel and the chain were at the level of 48.9 MPa, while the maximum equivalent stresses at the tooth base were at the level of 2.18 MPa. 

A total of 300 surface points were measured to determine the wear in the area of mating surfaces between the chain wheels and the chain links using a coordinate-measuring machine (detailed description of the method for determining the parameters characterizing the wear is presented in [26]). In the next step, the smallest distance δ_i,N_ between the i-th point of the measuring path—before and after the wear test—was determined. Then the results were averaged using the following relationship:

(3) $$\delta_{i\_\mathrm{AVG}}=\frac{\sum_{1}^{n}\delta_{i,N}}{n}$$

where *n*—the number of seat surfaces of a given chain wheel (*n* = 24).

On the basis of the determined wear parameters δ_i,N_, a single-figure indicator of maximum wear δ_MAX_ was calculated using the following relationship:

(4) $$\delta_{\mathrm{MAX}}=\frac{\sum_{1}^{n}\mathrm{Max}\left\{\delta_{i,N}\right\}}{n}$$

Table 4 presents the factor combinations that trigger destructive processes, used at the presented stage of testing.

## 3. Results and Discussion

**Figure 7:**
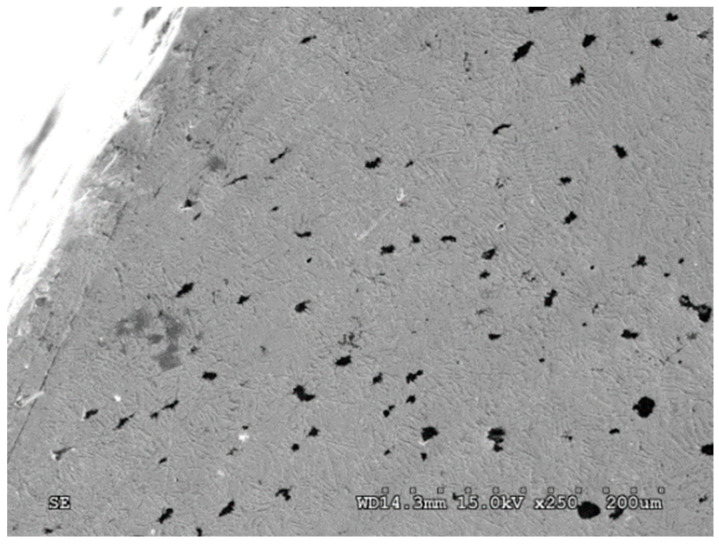
Deformation of the graphite in the surface layer of ADI_360 after abrasive wear (Variant A) (SEM; cross-section).

The phase composition of the microstructure of each variant of ADI had a significant impact on the matrix properties, including the wear intensity, and on the deformation manner of spheroidal graphite nodules, which was important for a development of the damage to the surface layer. In the case of cast iron with the structure of the upper ausferrite (ADI_360 and ADI_310) and characterized by a high content of austenite (27–40%), significant changes in the shape of the graphite nodules were observed. The degree of their deformation depended on the distance from the area of mating surfaces between the chain wheel seat and the chain link. The closer to the surface, the more decrease in the sphericity of graphite was found—over time the graphite took the form of narrow lenses arranged at an angle of 30–45° to the surface subjected to the action of the abrasive (Figure 7). In some cases, it was found that the graphite was arranged in parallel to the surface, which caused more extensive damage to the surface layer. Along with the reduction in the amount of austenite in the ADI microstructure, the area in which the deformed graphite occurred decreased; however, for ADI_240, no significant changes in the shape of the graphite took place under the load. Only traces of the graphite removed by the abrasive were observed for this ADI grade. 

On the surface of the chain wheels made of ADI subjected and not subjected to shot peening, there was a relatively small diversity of the mechanisms responsible for the destruction of the matrix in abrasive wear conditions. Micro-cutting of the ADI matrix by grains of the abrasive was the form of damage, revealed in the tested samples, i.e., for variant A and all the material combinations analyzed, there was no propagation of cracks deeper into the surface layer. 

**Figure 8:**
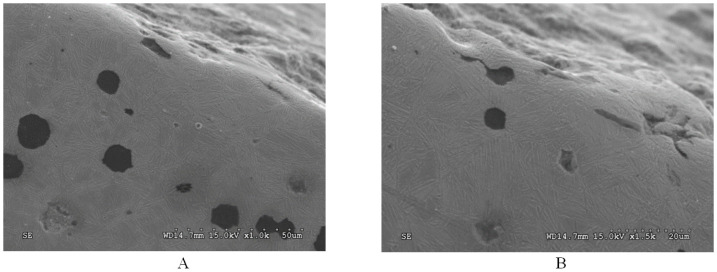
Deformations of graphite in the wheel made of ADI_360_D. (**A**) Graphite deformed as a result of the direct action of the abrasive at the point of contact between the socket and the chain. (**B**) Graphite with elongated and curved end deformation (SEM; cross-section).

In the case of ADI with the content of austenite in the range from 20% to 40% (both shot peened and not subjected to shot peening), which were tested in conditions of abrasive-dynamic wear (Variant B), it was observed, as in the previous variant, that the graphite was deformed as a result of the direct action of the abrasive at the point of contact between the seat and the chain (Figure 8A). However, the deformations had a smaller impact area than in the case of the abrasive wear by solid particles, which may indicate lower contact stresses that accompany cutting by abrasive grains. Figure 8B shows the characteristic appearance of a graphite nodule with a deformation in the form of an elongated and curved end, which may favor the separation of the cast iron matrix when cut by abrasive. No cracks in the surface layer were found for the cast irons discussed here.

**Figure 9:**
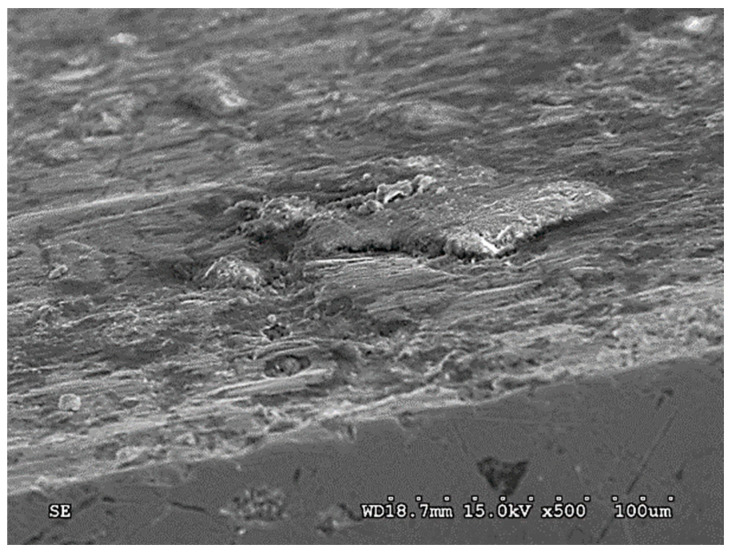
Cracks in the surface layer of the chain wheel made of ADI_240_D (SEM; cross-section).

**Figure 10:**
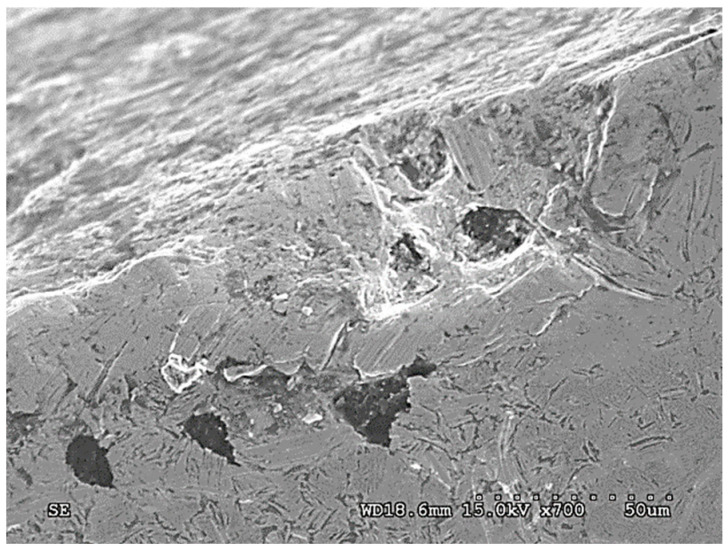
Chippings in the layer running along the boundaries of graphite nodules (ADI_240_D) (SEM; cross-section).

In the case of ADI_240_D, different forms of damage were observed—as compared with the abrasive wear variant. The following wear mechanisms were found in the surface layer of this cast iron:

- Micro-cutting of the surface by grains of quartz abrasive;
- Surface cracks of the surface layer (Figure 9);
- The action of the abrasive on the graphite nodules, resulting in the formation of large chippings of the surface (massive chippings of the surface layer running along the boundaries of the graphite nodules are shown in Figure 10).

**Figure 11:**
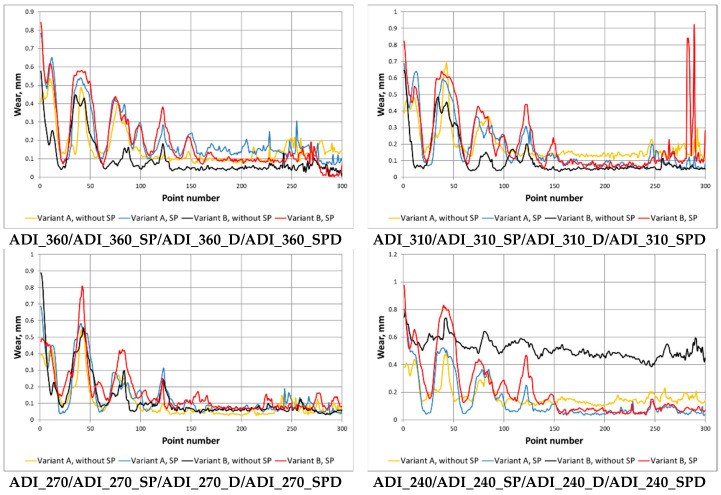
Waveforms of the averaged wear δ_i_AVG_ as a function of the position in the measuring path, determined for the considered ADI.

The last form of damage causes the largest cavities in the surface layer and may result from earlier cracks in the surface (such as in Figure 11) initiated by the action of dynamic forces. 

ADI_240 showed the highest susceptibility to the synergistic action of dynamic forces, which can be explained by the fact that ADI samples austempered at lower temperatures have the lowest resistance to cracking.

Figure 11 shows a comparison of the wear parameter δ_i_AVG_ in a function of the position in the measuring path (further in the paper this parameter will be denoted as δ_i_AVG_(i)), obtained for the abrasive wear variant and the abrasive-dynamic wear variant of all the grades of tested ADI.

**Figure 12:**
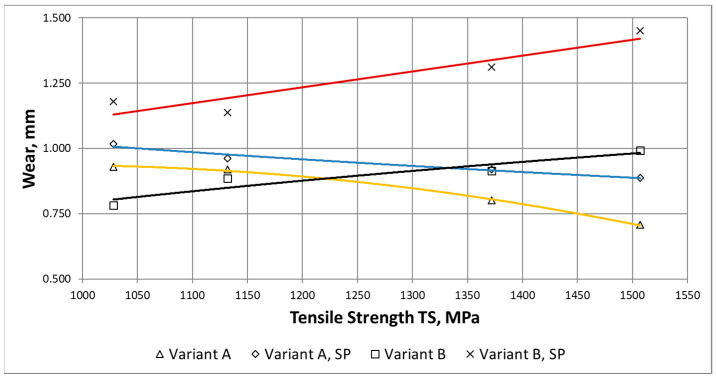
Waveforms of the wear parameter δ_MAX_ as a function of the maximum strength, determined for the variants of wear and dynamic treatment of the considered ADI.

Based on waveforms of the averaged wear δ_i_AVG_(i) in Figure 11, the wear parameter δ_MAX_ was determined. Figure 12 shows waveforms of δ_MAX_ as a function of the maximum strength for the ADI grades, surface condition and wear variants. 

On the basis of the waveforms presented in Figure 11 and Figure 12, it can be easily noticed that the combination of the abrasive factor with the external dynamic force increased significantly the total wear of the chain wheel seat bottom. The increase in the wear for the combination of destructive factors results from their synergistic action (this issue will be presented in more detail in the next section of the paper). 

In can be noticed in Figure 12, that along with the increase in the maximum strength (and also the surface hardness), the total wear of the ADI tested in the conditions of abrasive wear decreases (regardless of the type of surface treatment). For the case of the abrasive-dynamic wear, the relationship between the wear and the maximum strength was opposite. Shot peening of the area of mating surfaces of the chain wheels resulted in an increase in total wear.

For the variant of abrasive wear (Variant A), regardless of whether the wheels were subjected to shot peening or not, it can be stated that the highest wear resistance had ADI_240, while the lowest—ADI_360. The increase in the strength was accompanied by an almost linear decrease in the wear of the chain wheel surface. When analyzing the impact of the ADI making process (Figure 12), it can also be stated that an increase in the temperature of isothermal quenching (and the associated increase in the amount of austenite in the structure of the matrix of the tested ADI) leads to an increased wear.

**Figure 13:**
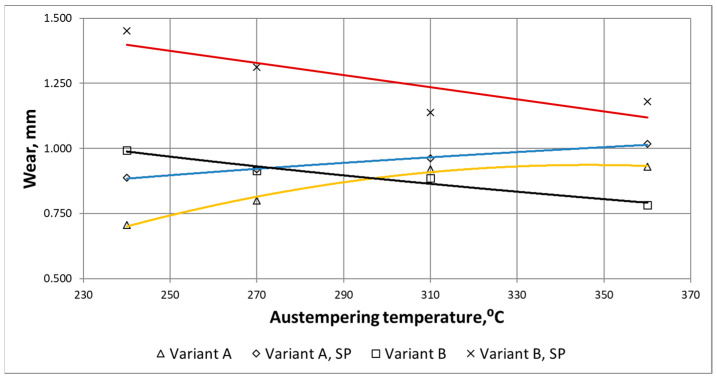
Waveforms of the wear parameter δ_MAX_ as a function of the isothermal quenching temperature of ADI, determined for different variants of wear and dynamic treatment.

In the case of the abrasive-dynamic wear test (Variant B) of the wheels not subjected to shot peening, the wheel made of ADI_360_D had the most advantages tribological properties. The next in this respect was the wheel made of ADI_310_D, while the wheel made of ADI_240_D had the lowest resistance to this type of wear. In the described case, it has been noticed that along with the increase in the austempering temperature and the content of austenite in the structure, the total wear of the mating surface of the wheel decreased (see Figure 13). In the case of the wheels subjected to shot peening, an identical relationship was obtained. 

The results obtained for the variant B are quite surprising because, as mentioned earlier, cast iron with the structure of the upper ausferrite usually shows worse wear resistance properties than cast iron with the structure of the lower ausferrite. When comparing the waveforms presented in Figure 13, a different impact of the austenite content on the wear resistance can be noticed. Different fracture toughness of each ADI variant (see Figure 9 and Figure 10) may be the reason for the different behavior of the ADI in the presence of the dynamic factor As mentioned earlier, the increase in the content of austenite in the ADI structure of causes an increase in the parameter K_IC_ characterizing the susceptibility of materials to brittle cracking. The obtained results allow stating that the wheels made of ADI with the structure of the upper ausferrite are more predestined for operation in conditions of abrasive wear by solid particles and variable action of dynamic forces.

**Figure 14:**
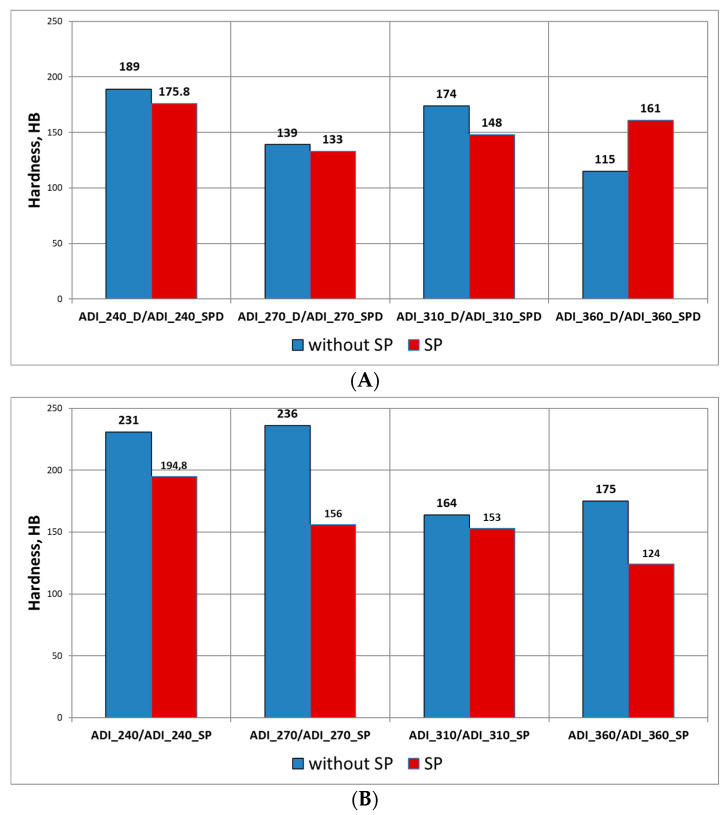
Change in the hardness of the operating surface layer in relation to the hardness of the technological surface layer: (**A**) after the wear test (Variant A), (**B**) after the wear test (Variant B).

After the wear tests, the hardness of the surfaces was measured. For all the variants of ADI, a significant increase in the hardness in relation to the hardness of the ADI before the tests (Figure 14) was found. The increase in the hardness of the operating surface layer in relation to the hardness of the technological surface layer is explained by the phase transformation of metastable austenite into martensite under the load and impact of hard quartz grains. As a result of the action of grains of the quartz abrasive, the initial hardness of ADI increased by 164–236 HB for the variant without shot peening, while for the variant with shot peening—by 124–194.8 HB.

**Figure 15:**
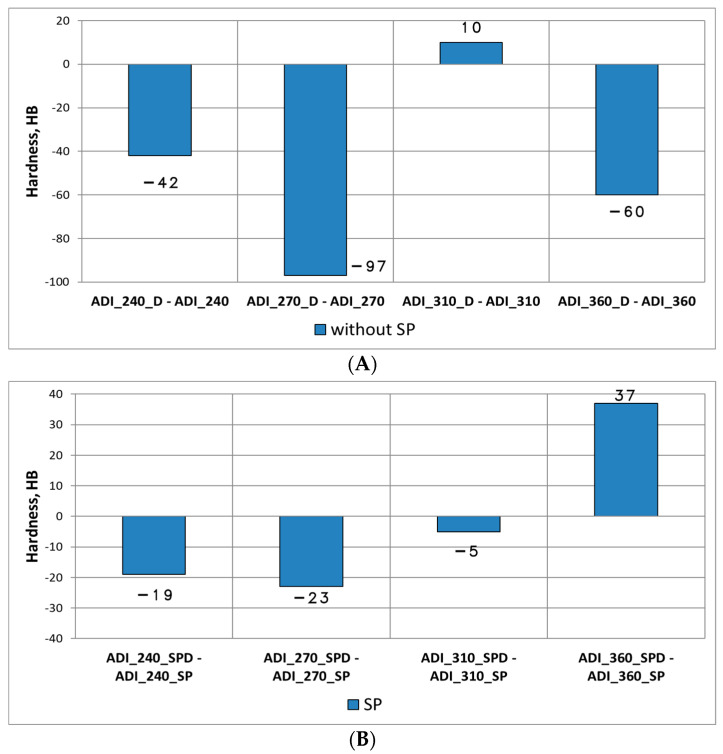
Difference in the hardness of the operating surface layer of wheels made of ADI, formed after the abrasive and abrasive-dynamic wear: (**A**) for the variant without shot peening, (**B**) for the variant with shot peening.

When comparing the results for both variants of wear (Variant A and B), in the majority of cases (Figure 15) a reduction in the hardness of the operating surface layer formed in conditions of abrasive-dynamic wear as compared with the hardness measured for the abrasive wear variant can be noticed. In particular, for the ADI not subjected to shot peening, except for the wheel made of ADI_310_D, after the abrasive-dynamic wear there was a noticeable decrease in the exploitation surface layer hardness in the range of 42–92 HB as compared with the hardness of the surface layer formed after the test in the presence of the abrasive only, while for the variant with shot peening the decrease in the hardness was 42–92 HB (except for the wheel made of ADI_360_SPD).

The decrease in the hardness of the operating surface layer may indicate for a reduced intensity of the phase transformation of austenite into martensite, which could be caused by a decrease in stresses generated when cutting the matrix and the resulting cracks in the surface layer due to the earlier combined action of the abrasive grains and dynamic load.

**Figure 16:**
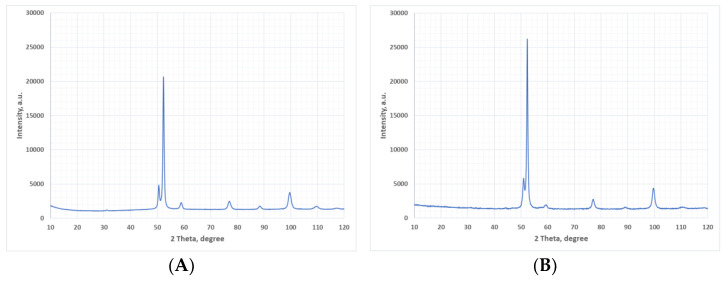
Examples of X-ray diffractograms for ADI_1200 cast iron: (**A**) initial state, (**B**) after wear test.

Figure 16 shows a comparison of the X-ray diffractograms determined for ADI_1200 cast iron at the initial state and after the abrasive wear test.

Diffraction measurements after the tribocorrosion test in industrial conditions were carried out in the contact zone of the sprocket tooth with chain links. Visible changes were found in the peak ranges identified with ferrite and with austenite. The increase in intensity should be explained by the formation of a martensitic structure as a result of the austenite phase transformation. According to the work [16], the diffraction peak of the martensite (110) resulting from the tetragonality of the elemental cell in the phase is superimposed on the strong diffraction line of the ferrite (110). Very similar results were obtained for the remaining material and operational variants

In the case of the wear tests, the interactive component ΔW_DYN_ (see: Equations (1) and (2)), resulting from the synergistic impact of the abrasive factor and external dynamic force on the process of wear of the chain wheel surface, can be determined as the difference between the combined abrasive and dynamic wear (factor W_T_ from Equations (1) and (2)), represented by the parameter δ_MAX,(B)_, and the abrasive wear alone, represented by the parameter δ_MAX,(A)_ (factor *W_ABR_* from Equations (1) and (2)). This is expressed by the following relationship:

(5) $$W_{T}=W_{ABR}+\Delta W_{DYN}$$

Similarly, the relationship of the synergistic component in a function of the position in the measuring path Δ*W_DYN_*,(i) will have the following form:

(6) $$\Delta W_{DYN,\left(i\right)}=\delta_{i_{\mathrm{AVG},\left(B\right)}}-\delta_{i_{\mathrm{AVG},\left(A\right)}}$$

**Figure 17:**
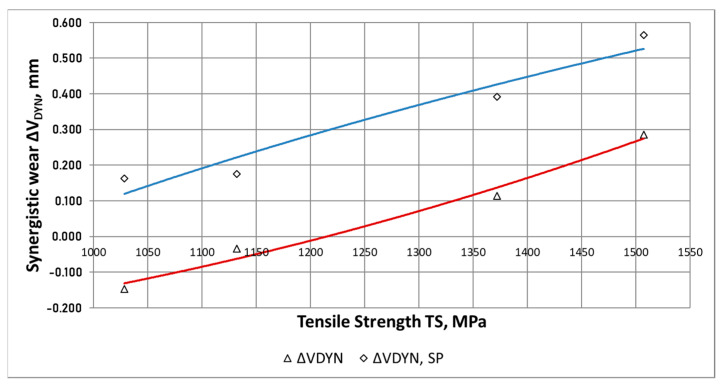
Waveforms of the component Δ*W_DYN_* as a function of the maximum strength of the tested ADI.

For the chain wheels not subjected to shot peening (ADI_360 and ADI_310), no synergistic effect of the action of the external force on the wear of the surface was found (Figure 15). It can be noticed that there is a rather small difference, not exceeding 15% (Figure 17).

The lack of synergistic effect in the case of the ADI_360 and ADI_310 wheels can be explained by a lower susceptibility to cracking of the ADI made at higher isothermal quenching temperatures. The effect of synergy between the degradation factors at the level of 20% was found for these shot peened cast iron samples.

A strong effect of synergy between the abrasive material and the external dynamic forces was observed in the case of chain wheels made of ADI_240 and ADI_270 with the structure of lower ausferrite. For the wheels not subjected to shot peening (ADI_240 and ADI_270), synergy effect was in the range of 14–34%, while for the shot peened wheels—in the range of 42–64%.

The synergism of degradation factors in the conditions of abrasive-dynamic wear can be explained by:

- An increase in micro-cutting of surface by the abrasive under additional dynamic forces;
- An increase in the number of microcracks caused by grains of the abrasive and their deeper propagation into the surface layer in the conditions of dynamic load acting on the friction pair.

**Figure 18:**
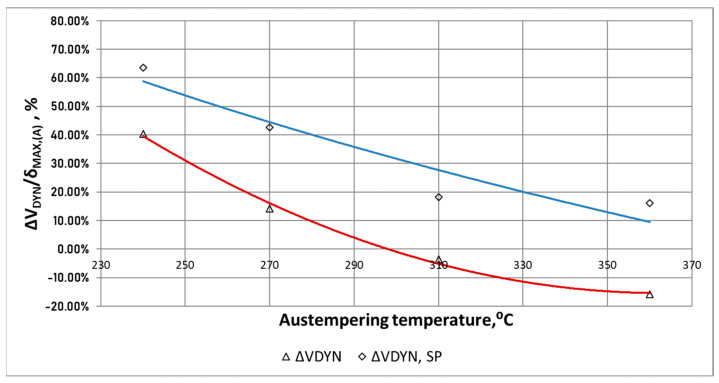
Waveforms of the relative increase in the wear Δ*W_DYN_*/δ_MAX,(A)_ as a function of the temperature of isothermal quenching of the tested ADI.

The relative increase in the wear Δ*W_DYN_*/δ_MAX,(A)_ (Figure 18), representing the share of the additional dynamic force in the process of wear, was in the range of 16–40% for the ADI not subjected to shot peening, while for the shot peened ADI—in the range of 16–64%.

## 4. Conclusions

Based on the tests results, the following detailed conclusions were formulated:

1. The impact of dynamic forces for chain wheels made of ADI containing Ni, Cu, Mo generally causes an increased degradation of the area of mating surfaces between the seat and the chain links.
2. Shot peening of the area of mating surfaces between the chain wheel and the chain resulted in an increase of the total wear of the mating surfaces area regardless of the variant of heat treatment of ADI.
3. The hardness of the surface layer of the shot peened chain wheels made of ADI decreased as compared with the variant not subjected to shot peening.
4. Along with the increase of the maximum strength and the simultaneous reduction in the content of austenite in the ADI structure containing Ni, Cu, Mo, an increase in the susceptibility to the synergistic effect, caused by the action of additional dynamic forces in the conditions of abrasive wear by solid particles, was observed.

## Figures and Tables

**Table 1:** Chemical composition of ductile iron (mass%), [28].

**C**	**Si**	**Mn**	**S**	**P**
3.50	2.54	0.16	0.013	0.041
**Mg**	**Cr**	**Cu**	**Ni**	**Mo**
0.047	0.026	0.50	1.40	0.24

**Table 2:** The process parameters used for manufacture of the tested ADI.

Heat Treatment Parameters *	ADI_240	ADI_270	ADI_310	ADI_360
Austenitizing temperature, °C	950			
Austenitizing time, min	180			
Austempering temperature, °C	240	270	310	360
Austempering time, min	150			

* Austenitizing and austempering temperatures and times were determined on the basis of earlier studies from [28].

**Table 3:** Mechanical properties of the tested ADI [28].

Mechanical Properties	ADI_240	ADI_270	ADI_310	ADI_360
Tensile Strength * TS, MPa	1507	1372	1132	1028
Yield Strength * YS, MPa	1072	936	804	652
Impact Toughness ** K, J	54	72	84	124
Elongation A5, %	3	4	5	10

* Values were obtained at stretching. ** Values are based on a notched Charpy sample.

**Table 4:** Combinations of damaging agents.

Research Variant and Its Designation	Destructive Factors *	Simulated Type of Wear
Variant A wheels not subjected to shot peening (-)	quartz sand	abrasive wear
Variant A wheels subjected to shot peening (SP)		
Variant B wheels not subjected to shot peening (D)	quartz sand and dynamic force	abrasive and dynamic wear
Variant B wheels subjected to shot peening (SPD)		

* Details related to destructive factors are included in [29].

## Data Availability

Not applicable.
